# Case report: Novel compound heterozygous variants in the *PANK2* gene in a Chinese patient diagnosed with ASD and ADHD

**DOI:** 10.3389/fneur.2023.1118076

**Published:** 2023-04-17

**Authors:** Siqi Dong, Ya Tuo, Zihan Qi, Yuanfeng Zhang, Xiaoni Liu, Ping Huang, Xiangjun Chen

**Affiliations:** ^1^Department of Neurology, Huashan Hospital and Institute of Neurology, Fudan University, Shanghai, China; ^2^National Center for Neurological Disorders, Shanghai, China; ^3^Department of Biochemistry and Physiology, Shanghai University of Medicine and Health Sciences, Shanghai, China; ^4^Department of Neurology, Shanghai Children's Hospital, Shanghai Jiao Tong University, Shanghai, China; ^5^Department of Forensic Pathology, Academy of Forensic Science, Shanghai, China; ^6^Human Phenome Institute, Fudan University, Shanghai, China

**Keywords:** PANK2 mutation, pantothenate kinase-associated neurodegeneration (PKAN), atypical PKAN, case report, autism spectral disorder (ASD)

## Abstract

The PANK2 gene, which encodes mitochondrial pantothenate kinase 2 protein, is the disease-causing gene for pantothenate kinase-associated neurodegeneration (PKAN). We report a case of atypical PKAN with autism-like symptoms presenting with speech difficulties, psychiatric symptoms, and mild developmental retardation. Magnetic resonance imaging (MRI) of the brain showed the typical “eye-of-the-tiger” sign. Whole-exon sequencing revealed *PANK2* p.Ile501Asn/p.Thr498Ser compound heterozygous variants. Our study highlights the phenotypic heterogeneity of PKAN, which can be confused with autism spectrum disorder (ASD) and attention-deficit hyperactivity disorder (ADHD) and requires careful clinical identification.

## Introduction

Mitochondrial pantothenate kinase 2 (PANK2) is a key regulatory enzyme in the biosynthesis of coenzyme A, which catalyzes the phosphorylation of pantothenate to generate 4′-phosphopantothenate. *PANK2* is the only one out of four *PANK* genes encoding an isoform that localizes to mitochondria. Homozygous or compound heterozygous variants of the *PANK2* gene can lead to pantothenate kinase-associated neurodegeneration (PKAN), the most prevalent type of neurodegeneration with brain iron accumulation (NBIA) disorders. PKAN patients present with a progressive movement disorder, dysarthria, cognitive impairment, and retinitis pigmentosa. In magnetic resonance imaging, PKAN patients exhibit the characteristic of the “eye of the tiger” sign in the globus pallidus, which corresponds to iron accumulation and gliosis as shown in neuropathological examinations (1).

Based on the age at onset and rate of disease progression, PKAN is classified into two major groups: (I) the classic form of PKAN characterized by early onset (<10 years old when first symptoms start), rapid progression, dysarthria, dystonia, rigidity, loss of ambulation ~15 years after the first symptoms, and pigmentary retinopathy and (II) atypical PKAN, which presents in the second decade of life, with slower progression, and heterogeneous phenotypes, such as extrapyramidal signs, neurobehavioral signs, and speech difficulties (2). Despite a series of reports, the phenotypic heterogeneity of PKAN is still not fully understood. Here we report a compound heterozygote for *PANK2* variants in a Chinese patient with early-onset atypical PKAN, which is easily confused with ASD.

## Case description

The patient, a 9-year-old boy, was recruited from a non-consanguineous family with no history of genetic diseases. His language and motor development were normal until he was 3 years old when his parents found that he rarely communicated or made eye contact with others. He did not catch up to their peers in language abilities. Psychiatric symptoms were also observed, including restricted or repetitive behaviors, emotional lability, attention deficit, and hyperactivity. At the age of 4, the patient gradually developed unstable walking, easy to fall, and often walked on tiptoes. He was taken to a local pediatric hospital and evaluated on a series of scales, including the Gesell test, Autism Behavior Checklist, psycho-educational profile, and Achenbach Child Behavior Checklist. The Gesell test suggested mild developmental retardation. The Autism Behavior Checklist scored 39. Psycho-educational profile suggested mild retardation in communication and moderate retardation in motor and maladaptive behaviors. Achenbach Child Behavior Checklist suggested significant social withdrawal. He was diagnosed with ASD and ADHD according to DSM-V criteria (persistent deficits in each of three areas of social communication and interaction plus two types of restricted, repetitive behaviors; eight symptoms for inattention and seven symptoms for hyperactivity and impulsivity). The patient thus underwent long-term rehabilitation behavior therapy, and the symptoms of unstable walking improved. At the age of 9, the patient's symptoms worsened after fever, characterized by frequent falls, tremors of the upper limbs, involuntary nodding of the head, and body shaking.

**Figure 1:**
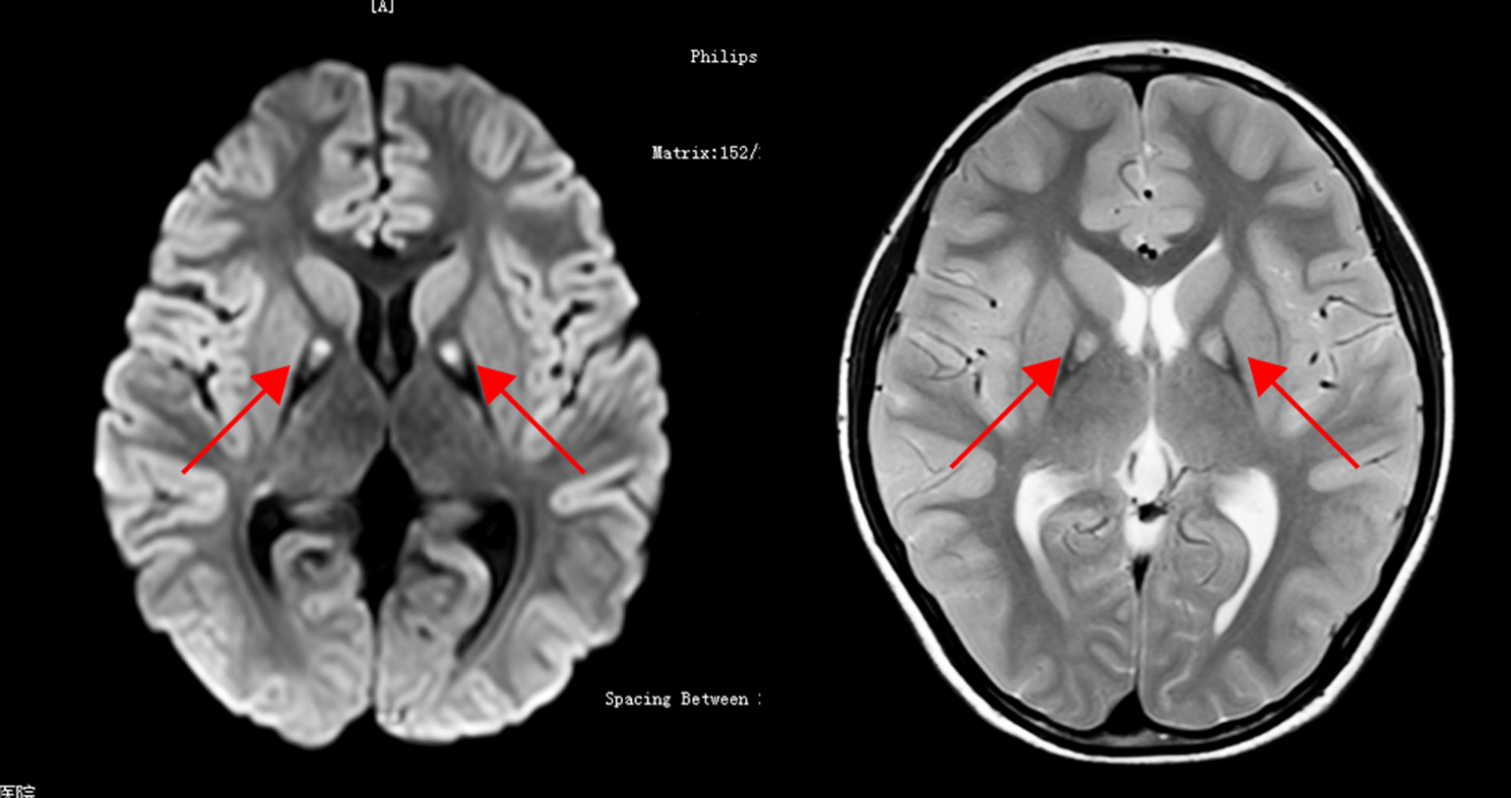
T2-weighted brain MRI showing the “eye-of-the-tiger” sign.

On examination, the patient showed involuntary nodding of the head, unstable walking, and was unable to complete the hoop. There was obstructed upward movement of both eyes and increased muscle tone, which is more prominent in the right limbs. Muscle strength and deep tendon reflexes were normal. Romberg signs and cerebellar signs were absent. Wechsler Intelligence Scale test was 70, which was at the borderline. Ophthalmologic examinations were normal except for slightly thinner retinal epithelial cells. Electroencephalogram showed no abnormalities. Laboratory tests, including routine blood and urine tests, biochemistry, liver and kidney functions, electrolytes, autoantibodies, and trace element tests were all normal. T2-weighted brain magnetic resonance imaging (MRI) showed a characteristic of the “eye-of-the-tiger” sign (Figure 1).

**Figure 2:**
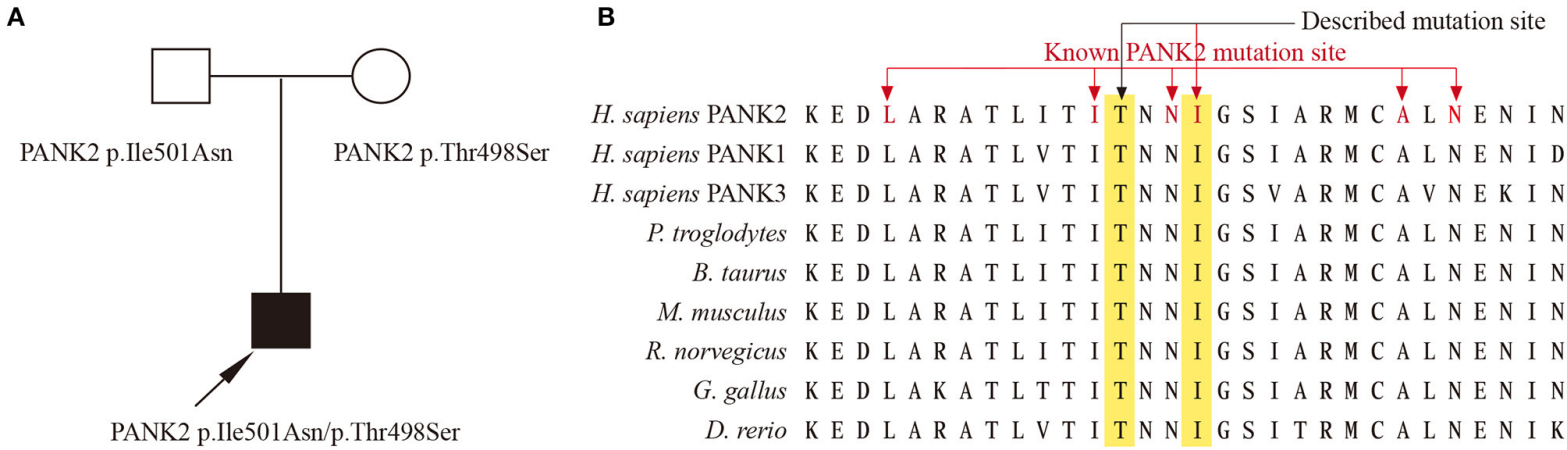
**(A)** Family with compound heterozygous PANK2 gene variants. Proband PKAN is indicated with an arrow. **(B)** Amino acid sequence alignment between species and PANK family proteins, the red font indicates amino acid changes previously reported in PKAN.

Whole-exon sequencing revealed two compound heterozygous variants (p.Ile501Asn and p.Thr498Ser) involving exons 5 in *PANK2* of the patient. The father was a heterozygote for the Ile501Asn variant, and the mother was a heterozygote for the Thr498Ser variant (Figure 2A). These two variants were absent in 1,000 g, ExAC, and gnomAD databases. *In silico* predictors, including SIFT, Polyphen2, and Variant Taster, predict Ile501Asn as “Damaging (0),” “Probably damaging,” and “Disease causing”; and Thr498Ser as “Damaging (0.004),” “Probably damaging,” and “Disease causing.” Amino acid sequence alignment shows that Ile501 and Thr498 are highly conserved both across species and in PANK family proteins (Figure 2B). Ile501Asn has been reported in several previous studies (2–4), while Thr498Ser was novel. According to the ACMG standard, *PANK2* p.Ile501Asn was classified as likely pathogenic (PS1, PM1, PM2, and PP3), while *PANK2* p.Thr498Ser was classified as a variant of uncertain significance (PM1, PM2, and PP3).

## Discussion

In this study, we report a case of early-onset PKAN in a patient presenting with atypical symptoms, including speech difficulties, psychiatric symptoms, and mild developmental retardation, mimicking ASD and ADHD. Whole-exon sequencing revealed PANK2 p.Ile501Asn/p.Thr498Ser compound heterozygous variants, of which Thr498Ser has not been reported earlier.

Despite having been diagnosed with ASD, a diagnosis of PKAN was more suitable in our patient since there was an “eye-of-the-tiger” sign in the T2-weighted brain MRI and the PANK2 compound heterozygous variant. The “Eye-of-the-tiger” sign is almost never excluded in PKAN patients, whether classic or atypical ones (2). Our patient was classified as atypical PKAN, as extrapyramidal signs, including dystonia, dysarthria, rigidity, and choreoathetosis, which is prominent in classic PKAN patients, were not severe and progressed slowly. While our patients present prominent speech difficulties, psychiatric symptoms, and mild developmental retardation, which were more common in atypical PKAN patients (2). Ile501Asn homozygous or complex heterozygous variants have previously been reported to cause atypical PKAN (2–4), consistent with our findings.

Interestingly, atypical PKAN is often present in the second decade of life, while our patient developed the disease at age 3, contrary to previous reports, making it easy to be confused with some neurodevelopmental diseases, such as ASD. By the revision of previous reports of PKAN in China (Table 1), we find that there are 42.9% of early-onset patients who showed slow progression, suggesting that some early-onset patients do not necessarily exhibit typical PKAN, consistent with our findings. Dystonia and dysarthria are the most common symptoms of PKAN, presenting in 77.4% of patients. Psychiatric symptoms, which are prominent in our patient, are rare, accounting for 6.5% by revision, and reported to be 9.1% in the previous literature (2). Therefore, PKAN patients with prominent psychiatric symptoms are rare, which requires careful identification. For children suspected of ASD and ADHD with poor sensory integration, MRI and genetic examination are needed to diagnose the disease at an early stage and give reasonable treatment.

**Table 1:** Clinical, radiological, and genetic characteristics in Chinese patients with PANK.

**Patient**	**Category**	**Onset age**	**Gender**	**Mutation1**	**Mutation2**	**Exon1**	**Exon2**	**Eye-of-the-tiger sign**	**Parkinsonism**	**Gait disturbance**	**Tremor**	**Dystonia**	**Dysarthria**	**Dysphagia**	**Retinal changes**	**Cognitive impairment**	**Psychiatric symptoms**	**Reference**
1	Atypical	3	M	p.T498S	p.l501N	Exon5	Exon5	Y	N	Y	Y	Y	N	N	N	Y	Y	Our patient
2	Atypical	20	F	p.E39X	p.D268G	Exon1	Exon3	Y	N	N	Y	N	Y	N	N	N	N	(5)
3	Atypical	25	M	p.D87G	p.H136fs	Exon1	Exon3	Y	N	Y	N	Y	Y	N	N	N	N	(6)
4	Atypical	3	F	p.L96fs	p.D161G	Exon1	Exon4	Y	N	N	N	Y	N	N	N	Y	N	(7)
5	Typical	6	M	p.M133V	p.M133V	Exon1	Exon1	Y	N	Y	N	Y	Y	N	Y	Y	N	(8)
6	Atypical	27	M	p.E149X	p.D378G	Exon1	Exon3	Y	Y	N	Y	Y	N	N	N	N	N	(9)
7	Atypical	20	F	p. E149X	p.D378G	Exon1	Exon3	Y	N	N	Y	Y	Y	N	N	N	N	(10)
8	Typical	Childhood	M	p.A170fs	p.R440P	Exon1	Exon4	Y	N	N	Y	Y	Y	N	N	Y	N	(11)
9	Typical	Childhood	M	p.A170fs	p.R440P	Exon1	Exon4	Y	N	N	Y	N	Y	N	N	Y	N	(11)
10	Typical	1	M	p.V172fs	p.G215D	Exon1	Exon2	Y	N	Y	Y	Y	Y	N	N	Y	N	(8)
11	Atypical	15	F	p.L210fs	p.F377S	Intron1	Exon3	Y	N	Y	Y	N	Y	Y	N	N	N	(12)
12	Typical	8	M	p.L210fs	p.F377S	Intron1	Exon3	Y	N	Y	Y	Y	Y	N	N	Y	N	(12)
13	Typical	4.5	M	p.D217G	p.D447E	Exon2	Exon4	Y	N	Y	Y	Y	Y	N	N	Y	N	(13)
14	atypical	17	F	p.D268G	p.I391N	Exon3	Exon5	Y	N	N	Y	Y	Y	N	N	N	N	(14)
15	Atypical	2	M	p.R286C	p.R286C	Exon2	Exon2	Y	Y	Y	N	N	Y	N	N	N	N	(7)
16	Atypical	7	M	p.R286C	p.R286C	Exon2	Exon2	Y	N	Y	N	Y	N	N	N	N	N	(7)
17	Atypical	3	M	p.R286C	p.R286C	Exon2	Exon2	Y	N	Y	N	Y	Y	N	N	N	N	(7)
18	Atypical	12	F	p.P288L	p.P288L	Exon2	Exon2	Y	N	Y	Y	Y	Y	N	N	N	N	(15)
19	Atypical	10	F	p. D324Y	p.D324Y	Exon2	Exon2	Y	N	Y	Y	Y	N	N	N	N	N	(10)
20	Atypical	18	M	p.T349X	/	Exon3	/	Y	N	N	Y	Y	Y	N	N	N	N	(16)
21	Typical	4	F	p. D368G	p.D368G	Exon3	Exon3	Y	N	Y	Y	Y	Y	N	N	Y	N	(10)
22	Atypical	22	M	p. D368G	p.N500I	Exon3	Exon5	Y	Y	Y	Y	N	Y	N	N	N	N	(10)
23	Atypical	Childhood	M	p.D378G	p.D452G	Exon3	Exon4	Y	N	N	Y	Y	Y	N	N	Y	N	(17)
24	Atypical	51	M	p.D378G	p.D452G	Exon3	Exon4	Y	N	Y	Y	Y	Y	N	N	N	N	(17)
25	Atypical	22	M	p.D378G	p.l501N	Exon3	Exon5	Y	N	N	Y	N	Y	N	N	N	N	(3)
26	Atypical	11	M	p.L387fs	p.L566V	Exon3	Exon7	Y	N	N	Y	Y	Y	N	N	N	Y	(14)
27	Typical	6	F	p. D452G	p.D452G	Exon4	Exon4	Y	N	Y	Y	Y	Y	N	N	N	N	(10)
28	Atypical	16	M	p. D452G	p.L566V	Exon4	Exon7	Y	Y	N	Y	N	Y	N	N	N	N	(10)
29	Typical	10	F	c.1413-13_1413-12insTTCCCC	/	Intron4	/	Y	N	Y	N	Y	N	N	Y	Y	N	(18)
30	Typical	7	F	p. R490fs	p.R490fs	Exon5	Exon5	Y	N	Y	Y	Y	Y	Y	N	N	N	(10)
31	Typical	11.5	M	p.F519L	p.Y536C	Exon6	Exon6	Y	N	Y	N	Y	N	N	N	N	N	(19)

In conclusion, our study highlights the phenotypic heterogeneity of PKAN, which can be confused with ASD and requires careful clinical identification.

## Data availability statement

The original contributions presented in the study are included in the article/supplementary material, further inquiries can be directed to the corresponding authors.

## Ethics statement

The studies involving human participants were reviewed and approved by the Ethics Committee of Huashan Hospital, Fudan University. Written informed consent to participate in this study was provided by the participants' legal guardian/next of kin. Written informed consent was obtained from the minor(s)' legal guardian/next of kin for the publication of any potentially identifiable images or data included in this article.

## Author contributions

Conception and study design: XC and PH. Data collection or acquisition: SD, YT, YZ, PH, and XC. Review of previous literatures: SD, ZQ, and XL. Drafting the manuscript work: SD, YT, PH, and XC. All authors contributed to the approval of final version to be published and agreement to be accountable for the integrity and accuracy of all aspects of the work.
